# Cesium salt of tungstophosphoric acid/mesoporous (zirconia–silica) composite for highly efficient synthesis of 7-hydroxy-4-methyl coumarin and removal of methylene blue†

**DOI:** 10.1039/d3ra02235h

**Published:** 2023-05-18

**Authors:** Amr Awad Ibrahim, Doaa A. Kospa, O. R. Hayes, A. S. Khder, S. A. El-Hakam, Awad I. Ahmed

**Affiliations:** a Department of Chemistry, Faculty of Science, Mansoura University Al-Mansoura 35516 Egypt amr_awad@mans.edu.eg +220502390551; b Chemistry Department, Faculty of Applied Science, Umm Al-Qura University 21955 Makkah Saudi Arabia

## Abstract

The removal of harmful organic dyes from aqueous solutions has drawn the attention of scientists because of the substantial threat they pose to society's worldwide health. Hence, it is crucial to design an adsorbent that is both very effective in removing dyes and has the benefit of being inexpensive. In the present work, Cs salts of tungstophosphoric acid (CPW) supported mesoporous Zr-mSiO_2_ (mZS) with varying extents of Cs ions have been prepared by a two-step impregnation technique. Accordingly, a lowering in the surface acidity modes was observed after Cs exchanged protons of H_3_W_12_O_40_ and formed salts immobilized on the mZS support. After exchanging the protons with Cs ions, the characterization results revealed that the primary Keggin structure was unaltered. Moreover, the Cs exchanged catalysts had higher surface area than the parent H_3_W_12_O_40_/mZS, suggesting that Cs reacts with H_3_W_12_O_40_ molecules to create new primary particles with smaller sizes possessing inter-crystallite centers with a higher dispersion degree. With an increase in Cs content and thus a decrease in the acid strength and surface acid density, the methylene blue (MB) monolayer adsorption capacities on CPW/mZS catalysts were increased and reached an uptake capacity of 359.9 mg g^−1^ for Cs_3_PW_12_O_40_/mZS (3.0CPW/mZS). The catalytic formation of 7-hydroxy-4-methyl coumarin was also studied at optimum conditions and it is found that the catalytic activity is influenced by the amount of exchangeable Cs with PW on the mZrS support, which is in turn influenced by the catalyst acidity. The catalyst kept approximately the initial catalytic activity even after the fifth cycle.

## Abstract 2

Cs salts of tungstophosphoric acid supported mZS with varying amounts of Cs ions content have been prepared by a two-step impregnation technique, and found to be effective for the removal of hazardous dyes such as MB from an aqueous solution.

## Graphical abstract

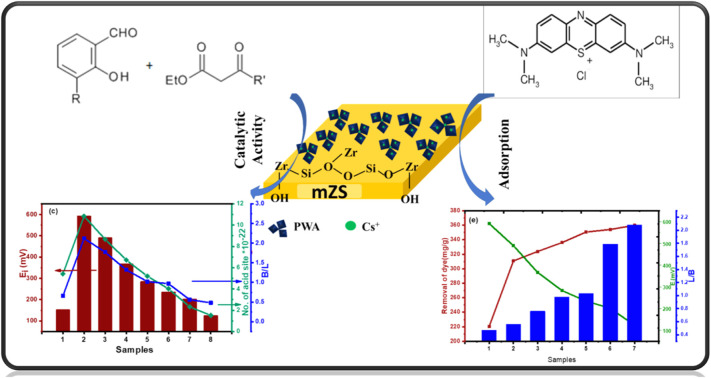

## 1. Introduction

Because of their ease of synthesis, acceptable stability, and extremely strong Brønsted acids, heteropolyacids (HPAs) have prompted a huge interest in acid-catalyzed processes.^1–3^ HPAs have been used in several industrial applications including the polymerization of tetrahydrofuran, hydration of *n*-butane and isobutylene, and the conversion of ethylene to acetic acid.^4–7^ Due to its highest acidity within the Keggin series, 12-tungstophosphoric acid (H_3_PW_12_O_40_) has been the target catalyst in several earlier studies.^8,9^ However, PW catalysts suffer from various limitations including limited thermal stability and solubility in polar solutions, and a small surface area (1–10 m^2^ g^−1^).^10^ To overcome these issues, acid salts of PW containing cations like NH_4_^+^, Cs^+^, Rb^+^, and K^+^ with surface areas of 150 m^2^ g^−1^ (ref. 11 and 12) can be prepared by supporting them on suitable supports such as mesoporous molecular sieves MCM-41,^13^ silica,^14^ silica-alumina,^15^ activated carbon,^16^ zeolites,^17^ metal–organic framework^18,19^ and other methods to increase surface area and, as a result, catalytic activity and longevity. To some extent, combining silica and zirconia enhances the surface area of zirconia while also improving its acidity and stability.^20^ Cesium-exchanged Keggin heteropolyacid salts with their various ratios (Cs_*x*_H_3−*x*_PW_12_O_40_) have been the most used catalysts due to their low solubility in aqueous and polar solvents compared with the Keggin structure of tungstophosphoric acid.^21,22^

**Scheme 1:**
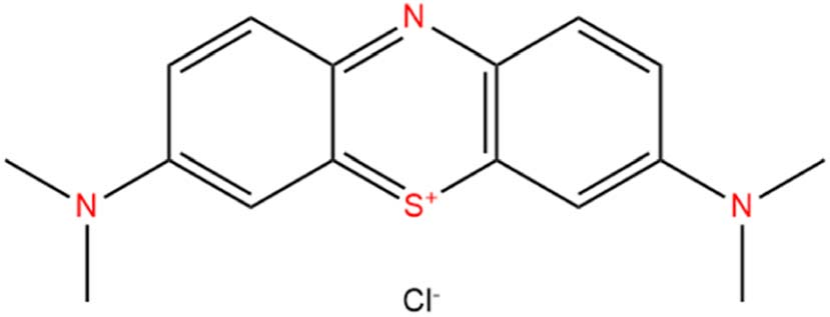
Methylene blue structure.

Water treatment has become a highly important subject in terms of environmental protection in recent years.^2^ Dye contamination is an increasing environmental hazard and efficient removal from water bodies is critical.^23,24^ With the rapid advancement of industrialization, more and more wastewater containing chemicals is discharged from diverse human activities, posing major environmental hazards.^24–26^ Methylene blue (MB), which is present in dyeing industry wastewaters, is a cationic dye, resistant to biodegradation, and stable in the presence of daylight and natural oxidizing agents.^27,28^ To dispose of wastewater, a variety of physicochemical procedures have been used, including electrocoagulation, photocatalytic process, flocculation, ozonation, adsorption, and membrane filtering.^28–33^ Because it is inexpensive, effective, and easy to modify, liquid-phase adsorption is the most efficient and cost-effective approach for treating contaminants in the water among the numerous purification procedures.^34^ Many different substances have been utilized as adsorbents to date for the removal of dyes from contaminated water, including carbon-based compounds, biomaterials, polymers, clays, and metal oxide nanoparticles.^35^ However, the majority of the adsorbents mentioned above have reached their practical applicability limits including the agglomeration in aqueous solution and poor adsorption capacity. Moreover, some of these adsorbents are effective for the removal of low concentrations of dye and they're selectively removing of the targeted organic dyes is still poor. Thus, it is extremely important to find a desirable adsorbent, which exhibits highly efficient removal of organic dyes with high selective separation.^36^ Methylene blue (Scheme 1) which causes different problems in human health is the most widely used cationic and basic dye in printing, textiles, biology, and chemistry. Zirconia–silicate (ZrSiO_4_) is highly insoluble in aqueous, acidic, and alkaline solutions and is widely used in heavy metal adsorption, surface cleaning, surface preparation of stainless steel parts, food industries, and other medical applications.^37^ Zirconia has high surface charges, the ability to form sterically stable varied coordinate compounds, and the capacity to accept lone pairs of electrons that increases its affinity towards sulfur and nitrogen donors in the MB structure bringing excellent adsorption abilities.^38^ On the other hand, Keggin hetero-polyacid and their salts have attracted intensive attention due to their rich topology and versatility, earth-abundant source, controllable shape and size, highly electronegative, and oxo-enriched surfaces.^39^ The incorporation of the Cs salt of phosphotungstic acid (polyoxometalate) with zirconia–silicate increases the number of oxygen atoms on the surface which increased the negative charge of the surface enhancing the electrostatic attraction with the cationic functional structure of the MB dye.^40^ Wherefore, Cs_*x*_H_3−*x*_PW/zirconia–silica composites are the potential and suitable adsorbent for selectively capturing cationic dyes.

**Scheme 2:**
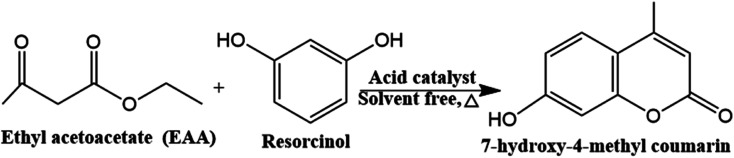
Pechmann reaction of 7-hydroxy-4-methyl coumarin.

Coumarins play a significant role in both synthetic and natural organic chemistry. Coumarins which found in a variety of natural materials and are commonly used as a starting material or intermediary in the pharmaceutical, fragrance, and agrochemical industries.^41^ It is also employed in the dispersed fluorescent, optical brighteners, production of anticoagulants, pesticides, and insecticides, as well as additives in cosmetics and food.^2,42,43^ Moreover, coumarins have been shown to exhibit a variety of bioactivities, including antibacterial, anticancer, and platelet aggregation inhibitory properties.^42^ Among the various coumarin derivatives, 7-Hydroxy-4-methyl coumarin has been used in several applications including fluorescent brightening, chemosensor, fluorometric determination of enzymatic activity, and the preparation of an insecticide (hymerocromone) and furanocoumarins.^43^ 7-hydroxy-4-methyl coumarin can be synthesized *via* various strategies and among all these strategies, the Pechmann reaction proceeds from very simple condensation of phenols and β-keto esters under acid catalysis giving good yields of the target coumarin derivatives (Scheme 2).^43^ Traditionally, the Pechmann reaction is catalyzed by homogenous acid catalysts including conc. H_2_SO_4_, AlCl_3_, P_4_O_10_, and CF_3_COOH, however, these catalysts yield by-products with the coumarin which require a long reaction time.^43^ Thus, the development of a cost-effective, facile synthesis of catalysts for the Pechmann reaction at milder reaction conditions with higher yields, is still a challenge. The heteropoly acids were successfully used as catalysts for this reaction due to strong acidic and redox sites, high catalytic performance, and selectivity to particular reaction products by selective stabilization of the produced intermediate through the reaction.^44^ H_3_PW_12_O_40_ as Brønsted acid has the highest acid strength and has higher acidity than traditional acids. The carbonyl group on ethyl acetoacetate can be chemisorbed on the Brønsted acid sites of the catalyst and the nucleophilic attack of resorcinol to form 7-hydroxy-4-methyl coumarin.^45^ However, the pure heteropolyacids were limited by their low stability in organic solvents, hence, heterogenous cesium-exchanged Keggin heteropolyacid salts with their various ratios (Cs_*x*_H_3−*x*_PW_12_O_40_) were modified to increase their stability. These salts can catalyze the Pechmann reaction through the Lewis acid sites originating from the metal cation which can accept lone pair of electrons from the carbonyl group of ethyl acetate as well as Brønsted acids generated from the Keggin structure.^45^ Finally, these salts are supported by zirconium silicate to increase the surface area of the catalyst.

Herein, we reported the facile and cost-effective synthesis of Cs_*x*_H_3−*x*_PW_12_O_40_/Zr-mSiO_2_ as high efficient catalyst for coumarin synthesis and adsorbent for cationic dyes removal. The cesium salt of the phosphotungstic acid was proposed in this study rather than the bare acid to prepare a low soluble catalyst or adsorbent even in organic solvents. Furthermore, the prepared catalysts were supported with the Zr/SiO_2_ to increase the surface area of these catalysts and the combination of silica and zirconia enhances the surface area of zirconia while also improving its acidity and stability. By using FT-IR, XRD TEM, pyridine adsorption, BET, and potentiometric titration, the structure and surface acidity of the fabricated catalysts were confirmed. As catalysts in the production of coumarin derivatives and effective adsorbents for the elimination of MB from aqueous solutions, *x* wt% Cs_*x*_H_3−*x*_PW_12_O_40_/Zr-mSiO_2_ composites were utilized. The prepared catalysts showed highly efficient catalytic activity and also high adsorption capacity and the activity increased with the increase of cesium content.

## 2. Experimental

### 2.1. Materials

As received, ethyl acetoacetate, *n*-butylamine, resorcinol, acetonitrile, pyridine, cesium nitrate (CsNO_3_), ammonium hydroxide (NH_4_OH), zirconium oxynitrate (ZrO(NO_3_)_2_), 12-tungstophosphoric acid (H_3_PW_12_O_40_:PW), methylene blue (MB), and sulfuric acid (H_2_SO_4_) were obtained from Alfa Aeser. Both cetyltrimethylammonium bromide (*N*-cetyl-*N*,*N*,*N*-trimethyl ammonium bromide, CTAB) and tetraethyl orthosilicate (TEOS) were analytical-grade reagents.

### 2.2. Preparation of catalysts

#### 2.2.1. Preparation of mesoporous zirconia–silica (ZrSiO_4_)

Tetraethyl orthosilicate (TEOS) and zirconium oxynitrate (ZrO(NO_3_)_2_) were used as the precursors to manufacture 15% zirconia on SiO_2_ (Zr/Si molar ratio of 0.7). Solution (A) was provided in the following manner: 15 wt% of ZrO(NO_3_)_2_ was mixed with 10 mL of TEOS till the complete dissolution and kept under vigorous stirring for three hours. Subsequently, about 2 g of CTAB as a surfactant template was dissolved in warm distilled water, added to 40 mL of aqueous NH_4_OH (32%), and kept for 30 min under stirring after the addition to produce a solution (B). After that, solution (A) was mixed dropwise with solution (B) while being vigorously stirred for two hours to produce ZrO(OH)_2_–Si(OH)_4_ gel. The final gel was put in a 150 mL Teflon-lined autoclave and heated at 80 °C for 48 h. The final product which had crystallized was centrifuged, washed with ethanol, and then distilled water and finally dried at 90 °C. The obtained solid was then annealed in the air (5 °C min^−1^) for 6 h at 550 °C to form the mesoporous zirconia–silica (mZS) sample.

#### 2.2.2. Preparation of Cs-tungstophosphoric acid/mesoporous zirconia–silica (Cs_*x*_H_3−*x*_PW_12_O_40_/ZrSiO_4_)

Highly dispersed Cs-tungstophosphoric acid salts on mZS support were prepared with different Cs content and were denoted as Cs_*x*_H_3−*x*_PW/mZS (*x* = 0.5, 1.0, 1.5, 2.0, 2.5, and 3). The samples were synthesized using a two-step impregnation procedure as follows; firstly, 1 g of the purely calcined mZS support was added to an aqueous solution of the 12-tungstophosphoric acid (PW) under vigorous stirring for 12 h at ambient temperature to produce 25 wt% H_3_PW_12_O_40_(PW) on (mZS), PW/ZS. After centrifuging, the final product was washed and evaporated for 10 h at 110 °C, and finally thermally annealed for 4 h at 450 °C. The Cs_*x*_H_3−*x*_PW_12_O_40_/mZS were synthesized by the incipient wetness technique where a solution containing certain amounts of CsNO_3_ was dropwise added to the PW/mZS suspension in water to exchange the proton of H_3_PW_12_O_40_ by Cs ions of CsNO_3_ while continues stirring of each solution overnight. After drying at 90 °C, the resultant catalysts were calcined for 4 h at 450 °C. The samples which were labeled as 0.5CPW/mZS, 1.0CPW/mZS, 1.5CPW/mZS, 2.0CPW/ZS, 2.5CPW/mZS, and 3.0CPW/mZS, were taken out of the furnace and put in a desiccator to prevent water from adhering to the sample surface. The abbreviations of samples and their chemical composition were listed in Table 1.

**Table 1:** The abbreviations of the as-prepared samples and their chemical composition

Abbreviation	Chemical composition
mZS	15% ZrO_2_–SiO_2_
PW/mZS	25% H_3_PW_12_O_40_/15% ZrO_2_–SiO_2_
0.5CPW/mZS	25% Cs_0.5_H_2.5_PW_12_O_40_/15% ZrO_2_–SiO_2_
1.0CPW/mZS	25% CsH_2_PW_12_O_40_/15% ZrO_2_–SiO_2_
1.5CPW/mZS	25% Cs_1.5_H_1.5_PW_12_O_40_/15% ZrO_2_–SiO_2_
2.0CPW/mZS	25% Cs_2_HPW_12_O_40_/15% ZrO_2_–SiO_2_
2.5CPW/mZS	25% Cs_2.5_H_0.5_PW_12_O_40_/15% ZrO_2_–SiO_2_
3.0CPW/mZS	25% Cs_3_PW_12_O_40_/15% ZrO_2_–SiO_2_

### 2.3. Materials characterization

With a PW150 Philips instrument of Ni-filtered Cu Kα radiation at 40 kV and a wavelength of 1.540, all prepared catalysts were evaluated by X-ray diffraction patterns (XRD) and scanned at 2*θ* from 1 to 70° Bragg's law^46^ was used to calculate the unit cell parameters *a*_0_ and *d*_100_ as shown in eqn (S1) and (S2).† Additionally, transmission electron spectroscopy (TEM) was applied at 120 kV by using a Jeol-Jem 1200EX II electron microscope. Before the TEM test, each sample was thoroughly dissolved in 1 mL of ethanol, and a drop of this dispersion was then put into a copper-grid-supported holey carbon sheet. The pore merits and sample surface areas were examined by using the high vacuum traditional volumetric glass technique. Before any adsorption measurements, the sample was first degassed using the BET technique for 4 h at 250 °C and a reduced pressure of 10^−5^ torr. Adsorption isotherms from BET measurements were demonstrated to measure the surface area.

The MATTSON-5000 FTIR spectrometer was demonstrated to evaluate the functional groups which existed in the fabricated catalysts in the 4000–500 cm^−1^ range with 128 scan and 4 cm^−1^ resolution, respectively. For the measurements and IR spectra recording, the material was ground into a powder using KBr and then pressed into a thin wafer. The total acidity of the surface of the prepared samples was calculated by using the potentiometric titration by 0.1 g of each sample dispersed in 10 mL of acetonitrile for 30 minutes and stirred for three hours.^22,23^ Afterward, by using an Orion 420 digital A model electrode, the dispersion was titrated against 0.025 N *n*-butylamine mixed with acetonitrile (0.005 mL min^−1^) to obtain the related potentials. Pyridine was adsorbed onto the catalyst surface, and its FT-IR spectra were acquired to investigate its Lewis and Brønsted acid sites. Before the pyridine adsorption, all the fabricated catalysts were stimulated to eliminate all water molecules or any molecules that had been adsorbed in the pores of catalysts at 250 °C in a vacuum. The fabricated catalysts were cooled down following degassing and then flashed dry pyridine in a vacuum for a month. The mixture was afterward evaporated to remove any extra pyridine, combined (5 mg) with 0.1 g KBr, and pressed in discs with a diameter of 30 mm to record the FT-IR spectra at wavelengths ranging from 1400–1700 cm^−1^.

### 2.4. Environmental applications

#### 2.4.1. Removal of methylene blue

Concerning the impact of several parameters (initial MB concentration, pH of the solution) on adsorption activity, a suspension that includes 0.1 g of Cs_*x*_H_3−*x*_PW_12_O_40_/mZS was combined while being stirred at 200 rpm with 25 mL of 150 mg g^−1^ MB solution in a flask submerged in a water bath that was held at 30 °C. At regular intervals, an aliquot of the solution was taken out and filtered by a glass fiber with a 0.7 m pore size. The amount of dye present in the filtrate was then conducted using a unicam 5625 UV/V spectrophotometer at *λ*_max_ of 662 nm at various periods.

The amount of MB dye adsorbing, *q*_e_ (mg g^−1^) by Cs_*x*_H_3−*x*_PW_12_O_40_/mZS samples was evaluated from the following eqn (1) and (2):1
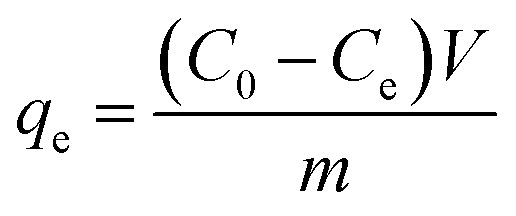
2
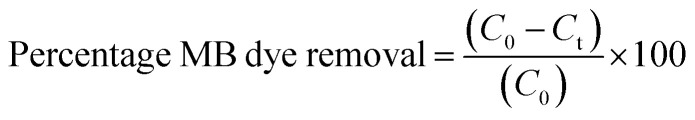
*C*_0_, *C*_e_, and *C*_*t*_ are respectively, designated the initial, equilibrium, and interval concentration of the MB dye (mg g^−1^), where *V* (L) denotes the MB solution volume and *m* (g) denotes the adsorbent mass.

#### 2.4.2. Synthesis of 7-hydroxy-4-methyl coumarin

By combining 10 mmol resorcinol with 20 mmol ethyl acetoacetate (EAA) and 100 mg active catalyst while stirring the mixture at 120 °C for 2 h, the coumarin derivative was prepared. In an ice bath, the hot mixture was transferred and stirred for about 15 min till the formation of 7-hydroxy-4-methyl coumarin precipitate which was centrifuged, washed, and evaporated at 80 °C to complete dryness. The product was stored and identified by IR measurements. According to the following equation, the % yield of the obtained 7-hydroxy-4-methyl coumarin was measured.3
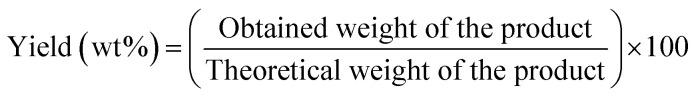


## 3. Results and discussion

### 3.1. Structural characterization

#### 3.1.1. FT-IR and XRD analysis

**Fig. 1:**
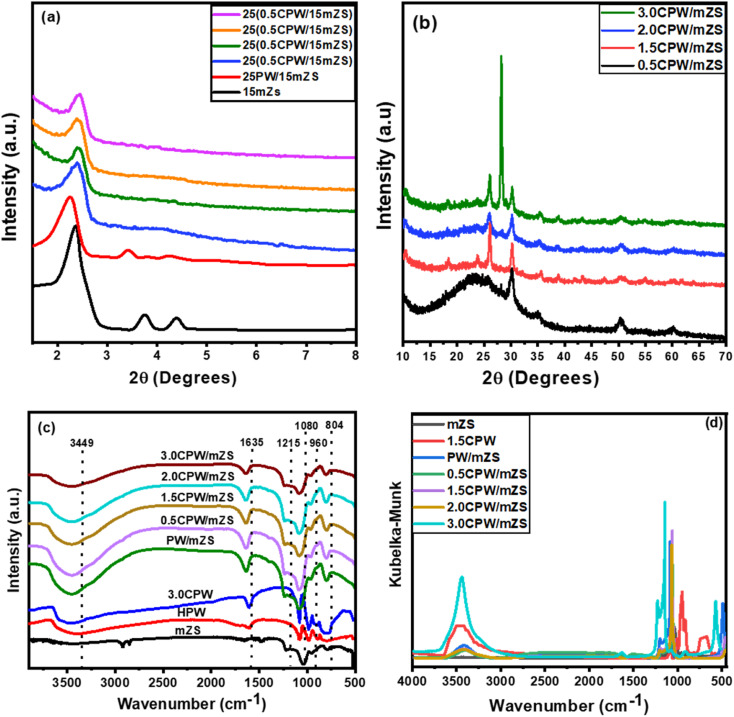
XRD diffractogram, (a) low-angle, and (b) high-angle patterns, and (c) FT-IR spectra, and (d) FT-IR spectra incoordinate Kubelka–Munk of mZS, HPW, 3.0CPW, PW/mZS, and Cs_*x*_H_3−*x*_PW_12_O_40_/mZS samples.

The XRD spectra of mZS, PW/mZS, and Cs_*x*_H_3−*x*_PW_12_O_40_/mZS samples were recorded and shown in Fig. 1. The figure shows that the mZS sample has a characteristic peak at 2*θ* of 2.4° (Fig. 1a) belongs to (100) reflection indicating that the samples possess the mesoporous framework structure, while the peaks at 2-theta of 3.8° and 4.4° related to (110) and (200) planes which are weakened and broadened on supporting PW on mZS, suggesting that the mesoporous ordering and crystallinity of mZS sample was decreased.^47,48^ The low of peaks at 2-theta of 3.8° and 4.4° related to (110) and (200) of mZS is due to the deformed-tetrahedral coordination of Zr in the framework was likely caused by the difference in ionic radius between Si and Zr (0.041 nm *vs.* 0.084 nm) since this has also been reported in other metal-containing mSiO_2_.^49,50^ Furthermore, Fig. 1a displayed the low-angle X-ray diffraction spectra for the different Cs_*x*_H_3−*x*_PW_12_O_40_/mZS catalysts. As shown in the figure, the peak at 2*θ* of 2.4° was observed, which corresponds to the (100) plane of a hexagonal unit cell. It was clear that Cs_*x*_H_3−*x*_PW_12_O_40_/mZS samples have a striking effect on the changes that occurred in the width and intensity of this reflection and the disappearance of the other two peaks indicating that the introduction of cesium ion to the samples reduced the order of the mesoporous structure. The hexagonal unit cell parameters *d*_100_, *a*_0,_ and *t* were calculated by eqn (S1) and (S2)† from the peak corresponding to (100) reflection in the XRD patterns. From the physicochemical properties in Table 1, by adding H_3_PW_12_O_40_, the values of the hexagonal unit cell parameter, *d*_100_, *a*_0_, and *t*, of mZS increased, indicating that pore size contraction was caused by PW film coating on the mesoporous channel of mZS. Moreover, the variation of the lattice cell parameters of Cs_*x*_H_3−*x*_PW_12_O_40_/mZS samples is related to the amount of incorporated Cs ions. The *d*_100_, *a*_0_, and *t* presented in Table 1 are more for Cs_*x*_H_3−*x*_PW_12_O_40_ loaded catalysts than pure mZS support but are less than the H_3_PW_12_O_40_/mZS sample indicating thus the introduction of Cs changes the position of H_3_PW_12_O_40_ on mZS surface. It is showed that the hexagonal order of the mesostructured materials strongly relied on the pores' texture properties^51^ which revealed that the pore size had contracted due to the lining of Cs_*x*_H_3−*x*_PW_12_O_40_ on the mesoporous channel of mZS. In addition, the high-angle XRD spectra of Cs-PWA/mZS samples were displayed in Fig. 1b. The figure showed four peaks with low intensity at 2*θ* of 18.4°, 23.8°, 35.6°, and 38.9° which are the characteristic peaks of the lines of the cubic PWA structure on Cs-PWA/mZS samples. However, sharp diffraction peaks at around 2*θ* of 25.4°, 26.1°, and 30.3° occurred for 1.5CPW/mZS, and 3.0CPW/mZS samples, and only one sharp peak at 2*θ* of 30.3° for 0.5CPW/mZS catalyst. These sharp peaks were attributed to the bulk crystal of the Cs_*x*_H_(3−*x*)_ PW_12_O_40_, up to three Cs^+^ ions per Kegging anion can be incorporated into the stable structure.

The FT-IR analysis was performed for the as-synthesized catalysts mZS, 3.0CPW, PW/mZS, and Cs_*x*_H_3−*x*_PW_12_O_40_/mZS and the obtained spectra and the spectra incoordinate Kubelka–Munk equation were represented in Fig. 1c and d, respectively. For mZS, the absorbed water has a peak at 3449 cm^−1^. While the stretching vibration of the –CH_3_ group from unhydrolyzed TEOS belongs to the peak at 2985 cm^−1^.^52,53^ Furthermore, the peaks of Si–OH and Si–CH_3_ groups of mZS were observed at 1635 and 1215 cm^−1^.^54^ Besides, the reported peak at 1050 cm^−1^ due to the Si–O–Si bond shifted to around 1080 cm^−1^ due to the partial replacement of Si atoms by Zr atoms breaking the symmetry of SiO_2_ and forming Zr–O–Si bonds.^52,55^ In case of pristine HPA and 3.0CPW before calcination, the primary structure of PW contains Kegging anions, which can be analyzed in the fingerprint region of 1700–600 cm^−1^ by FT-IR, which are at 3449 and 1610, 1080, 980, and 804 cm^−1^ which are attributed to OH of adsorbed water, P–O located in the central tetrahedra, W–O–W, and W=O vibrations, respectively.^56^ Moreover, for the calcined PW/mZS, and Cs_*x*_H_3−*x*_PW_12_O_40_/mZS, the spectra showed the characteristic peaks of mZs and Kegging anions with the small shifts indicating the successful combination between them. However, for Cs_*x*_H_3−*x*_PW_12_O_40_/mZS, the catalysts were distinctively characterized by the decrease of the W=O peak intensity,^56,57^ confirming the direct interaction between the Cs-salt and Keggin polyanion (Fig. S1†).

### 3.2. Nitrogen adsorption–desorption isotherms (BET measurements)

**Fig. 2:**
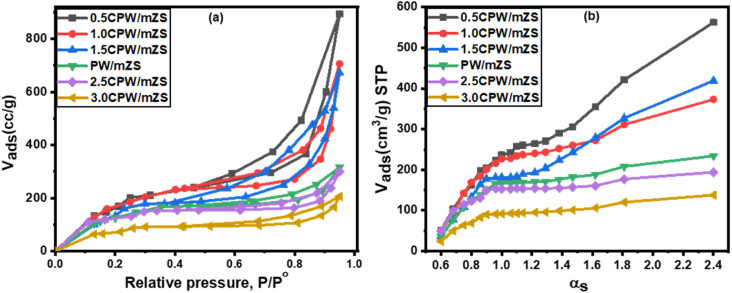
(a) Adsorption–desorption isotherms of nitrogen at −196 °C and (b) *α*_s_-plot for PW/mZS and Cs_*x*_H_1−*x*_PW_12_O_40_/mZS samples.

The BET measurements of PW/mZS, 0.5CPW/mZS, 1.0CPW/mZS, 1.5CPW/mZS, 2.5CPW/mZS, and 3.0CPW/mZS samples were recorded at a temperature of liquid nitrogen (−196 °C) and displayed in Fig. 2a. The obtained isotherms can be assigned to type IV isotherm, according to the IUPAC classification, that is characterized by three regions. Monolayer–multilayer adsorption on the pore walls accounts for increasing in N_2_ adsorption at low relative pressure, while capillary condensation within mesopores was responsible for the abrupt step at intermediate relative pressures. The multilayer adsorption on the adsorbent surface reaches a plateau at high relative pressures^58,59^ and the increase of the isotherm lines belongs to N_2_-condensation within voids of crystal aggregates. The homogeneity of the pore size is measured by the height and sharpness of the capillary condensation step which indicated that all as-prepared catalysts still possessed ordered mesopores structural with a narrow pore size distribution.

Table 1 demonstrated that the surface area reduced after loading PW on mZS support owing to the PW deposition into the mesochannels and on the surface of the mesoporous structure of mZS. On increasing the Cs content from 0.5CPW/mZS, 1.0CPW/ZS to 1.5CPW/mZS, the surface area is raised reaching 796, 742, and 709 m^2^ g^−1^ respectively. In such a situation Cs ions reacted with PW molecules that are located in the pores of the mZS support withdrawing them from the pores and also with that on the external surface forming primary particles with smaller sizes possessing the intercrystalline porosity in the salts,^61^ which were created by the rotation or translation of the Keggin anions during the ion exchange process as reported in TEM. The increase of Cs content above 1.5 caused a sharp reduction in the surface area which is related to the aggregation that appeared in TEM images. The above discussion confirmed that at low Cs content, the replacement of Cs by the protons significantly enhanced the porosity and relative surface area of the materials, this expiation is online with the results of XRD analysis and the values of *d*_100_, *a*_0_, and *t* of Table 2.

**Table 2:** Physicochemical characteristics of Cs_*x*_H_3−*x*_PW_12_O_40_/mZS samples

Samples	*d*_100_ (nm)	*A*_0_ (nm)	*t* (nm)	*S*_BET_ (m^2^ g^−1^)	*S*_s_ (m^2^ g^−1^)
mZS	3.26	3.78	1.07	726	713
PW/mZS	3.53	4.09	1.19	472	472
0.5CPW/mZS	3.42	3.96	1.13	796	787
1.0CPW/mZS	—	—	—	742	709
1.5CPW/mZS	3.38	3.91	1.12	709	701
2.0CPW/mZS	3.33	3.89	1.11	523	515
2.5CPW/mZS	—	—	—	341	335
3.0CPW/mZS	3.29	3.83	1.10	313	303

Moreover, the *α*_s_-plots which displayed the distribution of the different pores on the sample surface were given in Fig. 2b. This figure showed different observations; a straight line extends from the origin to *α*_s_ 0.9 corresponding to the complete monolayer adsorption with a slope which was utilized to measure the sample-specific surface area (*S*_s_).^60^ The type of the pores affects how *α*_s_-plots deviate from linearity; a pronounced upward departure at large film thickness and a positive intercept indicated the presence of small micropore volume and dominant mesoporosity. The flat linear portion of the curves may arise from the surface multilayer adsorption. Moreover, the observed low slope of the linear portion of *α*_s_-plots owed that the mesopores have been satisfied and the N_2_ adsorbed on the external sample surface. The orders of the arrangement of the estimated values of *S*_BET_ agree with that of *S*_s_, which revealed the validity of both methods for the surface area estimation.

### 3.3. Surface morphology

**Fig. 3:**
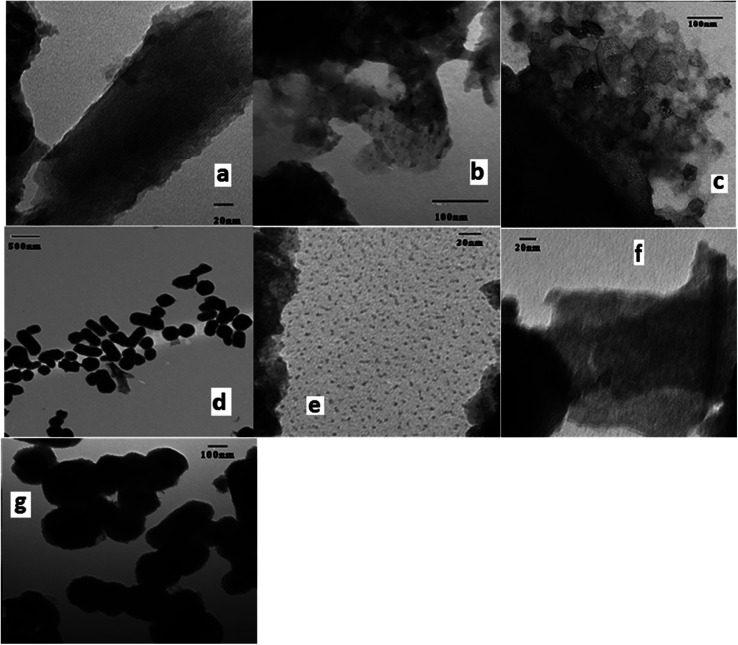
TEM images of (a, b) mZS (c) PW/mZS (d, e) 0.5CPW/mZS, and (f, g) 2.0CPW/mZS samples.

TEM images mZS, Fig. 3a and b illustrates an ordered structure with homogenous mesopores organized into a hexagonal, honeycomb-like lattice. The mesopores are not necessarily running in a straight way through ZS matrix, but they can be slightly curved, thereby retaining the hexagonal ordering, as can be seen in the figure. It appears that Zr particles have a wide average diameter of 8.2 nm causing the decrease in the mesoporous order structure of the silica. On the other hand, the TEM image of PW/mZS Fig. 3c shows the aggregation of PW and forming of large dark spots on the surface because of the formation of multilayer of PW on mZS surface and the mesoporous ordering of the ZS is decreased with the introduction of PW ions to ZS sample. The TEM image of 0.5CPW/mZS showed the existence of excellent distribution of Cs particles of PW salt on mZS particles with an average size range of 1.5–2.0 nm, this is can be noticed by the small dark spots on the surface because of the dispersion of Cs particles of 0.5CPW/mZS on the surface of mZS,^61^Fig. 3d and e. For 2.0CPW/mZS in Fig. 3f and g, showed large spots which refer to large Cs particles of 2.0CPW/mZS due to the aggregation of small Cs cores.^62^ Besides, 2.0CPW/mZS deposits can partially block the mesopores of the structure in line with the drop in surface area.

### 3.4. The surface acidity

Using *n*-butylamine (p*K*_a_ = 10.73), a basic molecule for the titration of the strong and medium acid sites on the surface of the catalyst in a nonaqueous solution for the surface acidity of the catalyst's determination. According to Pizzio *et al.* suggestion, potentiometric titration can be demonstrated to assess the catalyst acidity parameters, as well as the acidic strength and the total number of acid sites from the steady point of the plateau of the potentiometric titration curve as follows:^63^4
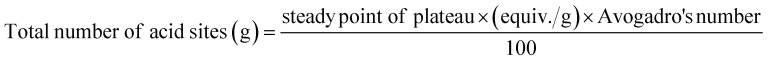


**Fig. 4:**
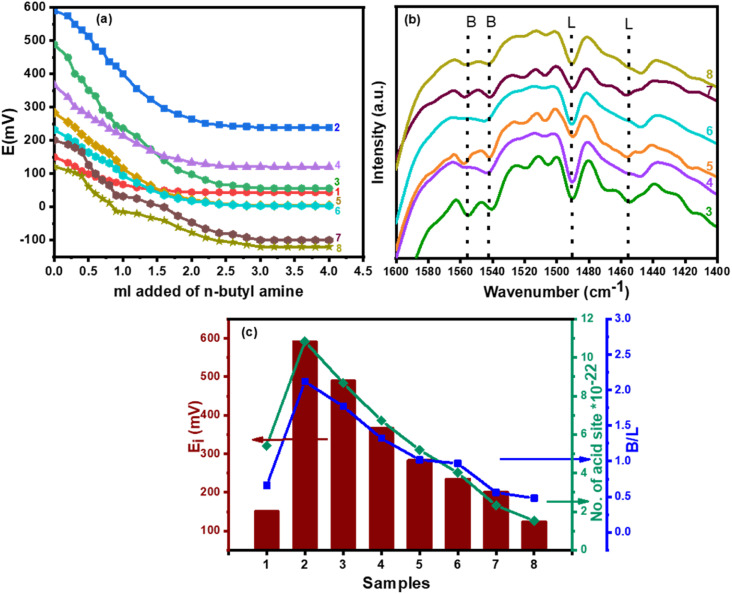
(a) Potentiometric titration, (b) FTIR spectra of the pyridine adsorbed on the surface, and (c) *E*_i_, active sites, and B/L ratio of (1) mZS, (2) PW/mZS, (3) 0.5CPW/mZS, (4) 1.0CPW/mZS, (5) 1.5CPW/mZS, (6) 2.0CPW/mZS, (7) 2.5CPW/mZS, (8) 3.0CPW/mZS.

The initial electrode potential (*E*_i_) of this approach reflects the maximal acid strength of the surface sites and the range that a plateau is established (*m*_eq_/g solid) revealing the total number of acid sites.^64^Fig. 4a shows the acidity result of catalysts calculated under investigation and the calculated values were reported in Table 2. It was shown that the acid strength and the total acidity (surface acid density/m^2^) of CPW/mZS catalysts are decreased by the increase of Cs content. This is because the catalysts lost most of their Brønsted acidity due to the substitution of the PW protons with cesium atoms.

The fingerprint, Keggin bands, of Cs_*x*_PWA/mZS, does not alter when it is exposed to pyridine as shown in Fig. 4b. From 0.5CPW/mZS to 2.5CPW/mZS, the band at 1540 cm^−1^ decreased in intensity until it nearly dissipates for Cs_3_PWA/mZS. On 3.0CPW/mZS, the small band at 1488 cm^−1^ faded away, which may likewise be attributable to the presence of pyridinium. Thus, the residual protons present on 3.0CPW/mZS give rise exclusively to hydrogen-bonded acid sites, while 1.0CPW/mZS, 2.0CPW/mZS, and 2.5CPW/mZS have Brønsted and hydrogen-bonded sites. By identifying dominant acid-type species present, which is typically expressed as the B/L ratio and measured from the FT-IR peak intensities of the pyridine-Bronsted and pyridine-Lewis acid site complexes at 1540 cm^−1^ and 1445 cm^−1^, respectively. According to Table 2, which showed the ratio of Brønsted to Lewis acid sites, the ratio decreases as the cesium level rises due to the increased Lewis acidity which was related to the decrease in the protons available to form the pyridinium ion (Brønsted acid sites) and the increase in coordinated pyridine (Lewis acid sites) related to the increasing of Lewis acid centers.^65^ The decreased amount of acid site ratios going from PW/mZS > 0.5CPW/mZS > 1.0CPW/mZS > 1.5CPW/mZS > 2.0CPW/mZS > 2.5CPW/mZS > 3.0CPW/mZS, indicated that exposure of protons is markedly independent of the surface area for these Cs_*x*_H_3−*x*_PW_12_O_40_ compounds as we discussed on the surface area measurements. The decline in the surface density and acid strength (*E*_i_) of Cs_*x*_H_3−*x*_PW_12_O_40_/mZS samples are other reasons that probably confirmed the aggregation of Cs_*x*_H_3−*x*_PW_12_O_40_ on mZS as also evident from TEM. Both methods of assessing acidity (Table 3) revealed a similar pattern of catalyst acidity arrangement, although the electrode potential measured both the acid strength and total acidity, whereas the pyridine adsorption was demonstrated to distinguish between the Brønsted and Lewis acid site types (Fig. 4c).

**Table 3:** Effect of Cs content on the physicochemical properties of Cs-PW/mZS samples

Catalysts	*E*_i_ (mV)	Surface acid density/m^2^ 10^−19^	B/L	L/B	Removal of MB *q*_exp_ (mg g^−1^)	7-Hydroxy-4-methyl coumarin (wt%)
PW/mZS	590.8	22.96	2.12	0.47	220.4	97.1
0.5CPW/mZS	489.4	10.89	1.77	0.56	310.8	82.6
1.0CPW/mZS	366.3	9.06	1.32	0.76	323.8	68.2
1.5CPW/mZS	283.2	7.34	1.02	0.98	336.1	46.6
2.0CPW/mZS	234.6	7.69	0.97	1.03	350.6	33.2
2.5CPW/mZS	201.2	6.81	0.56	1.79	354.1	22.3
3.0CPW/mZS	123.9	4.85	0.48	2.08	359.9	15.2

### 3.5. Environmental applications

#### 3.5.1. Free solvent synthesis of 7-hydroxy-4-methyl coumarin

##### 3.5.1.1. Optimum conditions of catalytic synthesis

**Fig. 5:**
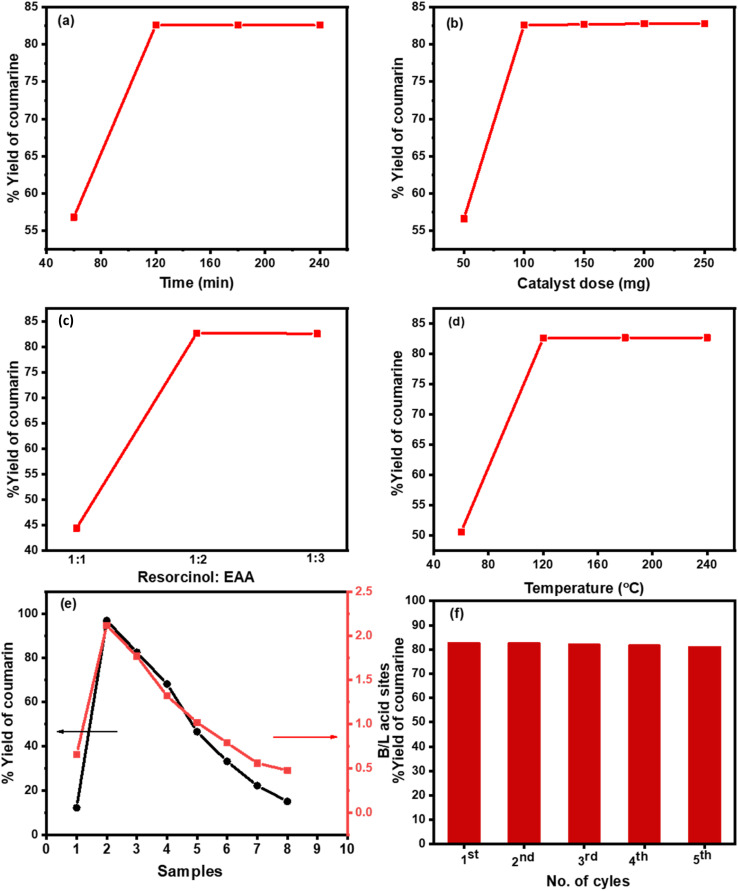
Effects of (a) time, (b) catalyst dose, (c) molar ratio of reactants, (d) temperature of 0.5CPW/mZS, (e) %yield of coumarin and B/L ratio of (1) mZS, (2) PW/mZS, (3) 0.5CPW/mZS, (4) 1.0CPW/mZS, (5) 1.5CPW/mZS, (6) 2.0CPW/mZS, (7) 2.5CPW/mZS, (8) 3.0CPW/mZS, and (f) reusability of 0.5CPW/mZS catalyst on coumarin synthesis.

Cs-PWA/mZS catalysts were used for the condensation reaction of resorcinol with ethyl acetoacetate in free solvent conditions. By adjusting the reaction duration, catalyst quantity (0.5CPW/mZS), molar ratio, and temperature conditions, the maximum conversion of the reactants to 7-hydroxy-4-methyl coumarin was obtained. The reaction duration varied from 60 to 240 min during the manufacture of 7-hydroxy-4-methyl coumarin (Fig. 5a) and the highest conversion of 82.6% with 100% selectivity was obtained in 120 min which is comparable to the reported studies (Table 4). Further increasing the reaction time had no impact on conversion or selectivity, which could be the result of active sites becoming inactive or the effective concentration of the reactants being lower. Additionally, the 7-hydroxy-4-methyl coumarin yield is significantly influenced by the amount of catalyst present in the reaction mixture, so the catalyst quantity of 0.5CPW/mZS has changed to 50, 100, 150, and 250 mg (Fig. 5b). With 100% selectivity, conversion was 56.6% at 50 mg of catalyst, and it raised to 82.6% at 100 mg of catalyst. No discernible change in the conversion was seen as the catalyst concentration was increased further because the catalyst was dispersed more evenly throughout the reaction system and the active acid sites on the catalyst were more readily available to the substrate. As a result, the conversion enhanced and reached its maximum level. Besides, various molar ratios of the reactants were utilized in the process (1 : 1, 1 : 2, and 1 : 3 of resorcinol : EAA) and it was observed that the amount of ethyl acetoacetate enhanced the yield to 44.4, 82.7, and 82.6%, respectively (Fig. 5c). The ratio of 1 : 2 resorcinol to EAA exhibited the optimum ratio for the solvent-free reaction and further increase in the ratio of the reactants produced the same %yield. The catalytic activity of the Pechman reaction was examined at the optimum conditions using 0.5CPWZS catalyst at different temperatures from 60 to 280 °C (Fig. 5d). It was found that the product increased gradually from 50.7 to the maximum value of 82.6% with 100% selectivity for 7-hydroxy 4-methyl coumarin at 120 °C. Further increase in temperature no significant change in the conversion was observed.

**Table 4:** Comparison study for synthesis of 7-hydroxy-4-methyl coumarin in other catalysts reported in the literature with this work

Catalyst	Conditions	Yield (%)	Ref.
PMA/Cr-Mg-MOF	Free solvent, reflux, 120 °C, 2 h	68.7	18
Nano Au@porous SiO_2_	Free solvent, reflux, 130 °C, 25 min	95	66
HClO_4_·SiO_2_	Free solvent, reflux, 130 °C, 90 min	62–98	67
Fe_3_O_4_@SiO_2_–BSA	Free solvent, reflux, 100 °C, 90 min	90	68
Sulfated zirconia	Free solvent, reflux, 130 °C, 20 min	78–85	69
PVP-supported H_3_PW_12_O_40_	Free solvent, reflux, 110 °C, 2 h	96.7	43
60% HPW/MCM-41	Free solvent, reflux, 120 °C for 1 h	97.1	63
H_3_PW_12_O_40_	Free solvent, reflux, 80 °C, 30 min	88	70
H_6_P_2_W_18_O_62_	Free solvent, reflux, 90 °C, 3 h	82	71
**0.5CPW mZS**	**Free solvent, 120 °C, 2 h**	**82.5**	**This work**

Using the recorded optimum conditions and in the comparison of the strength of acid sites (*E*_i_), surface acid density/m^2^ (the number of acid sites g^−1^/*S*_BET_, m^2^ g^−1^), and the B/L acid ratio of 0.5CPW/mZS to 3.0CPW/mZS, Fig. 4e and Table 2 and it was noted that when the surface acidity decreased, the catalytic activity of these catalysts consequently decreased. Moreover, the activity of the Cs-PWA/mZS catalysts continuously decreased when increasing the Cs content from 0.5CPW/mZS to 3.0CPW/mZS suggesting that the acid strength, B/L ratio, and the number of acid sites are the main factors in determining the catalytic activity towards Pechmann reaction of ethyl acetoacetate and resorcinol. Finally, the catalyst reusability was performed for the Pechmann reaction (Fig. 5f) under optimized reaction conditions using 0.5CPW/mZS. After the reaction was finished, the mixture was filtered and then washed several times with acetone to remove the entire reaction residue. After the activation of the recovered catalyst, the same reaction was repeated, and the catalyst was isolated after each reaction cycle for reusing. Fig. 5f displayed that the 0.5CPW/mZS produced smoothly a yield of 82.6–80.8% which showed that at least four cycles of the same activity could be achieved by reusing the catalyst.

##### 3.5.1.2. Mechanism of the catalytic reaction

To synthesize 7-hydroxy-4-methyl coumarin using the condensation reaction of Pechmann, two main molecular routes have been suggested in the literature.^44,67^ According to one study, ethyl acetoacetate (EAA) reacts with the resorcinol in a condensation synthesis process with the presence of an acidic catalyst to yield 7-hydroxy-4-methyl coumarin as shown in eqn (5). The proposed mechanism of this reaction is as follows; the EAA interacts with the catalyst, followed by the nucleophilic attack of the hydroxyl group on the resorcinol, results in the proton transfer from the acid sites of the catalyst to the keto group of EAA, which produces an intermediate and ethanol. The intermediate rapidly cycled through an acid-catalyzed intramolecular condensation process to provide 7-hydroxy-4-methyl coumarin. The electrophilic reaction of chemisorbed ethyl acetoacetate on resorcinol, which results in the production of chromones as a byproduct, is based on another potential mechanism. The mechanism suggested in the original study was ruled out because we did not see the synthesis of chromones during the current reaction. The potential mechanism for the synthesis of 7-hydroxy-4-methyl coumarin utilizing synthetic Cs-PWA/Zr-mSiO_2_ catalysts was suggested and is shown in Scheme 3.5
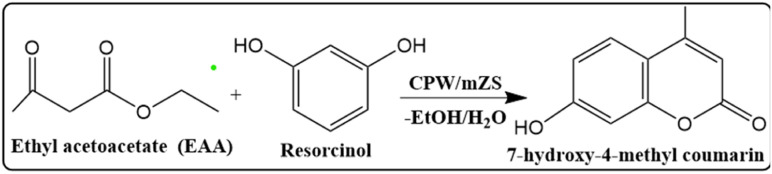


**Scheme 3:**
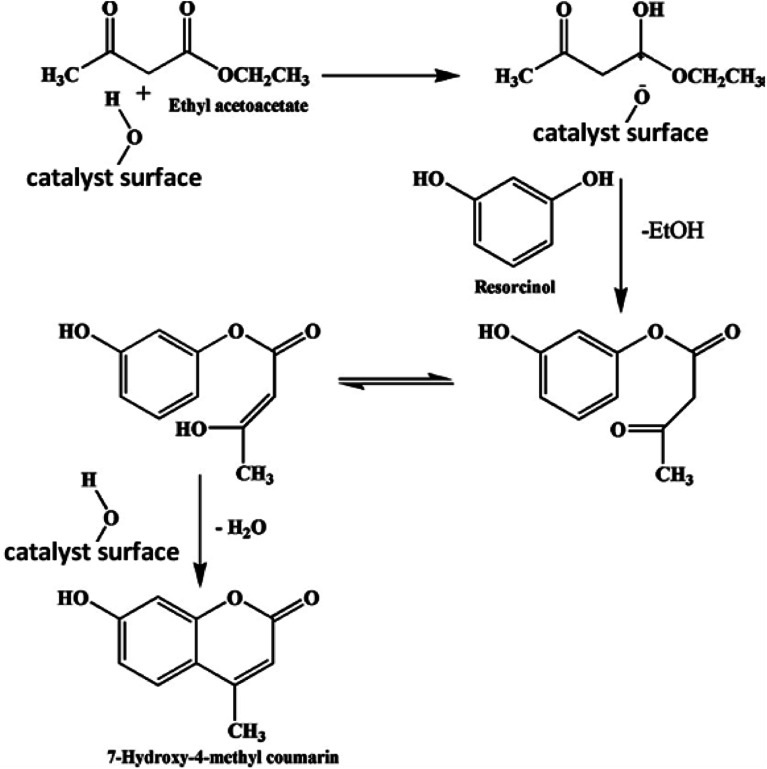
Mechanism of catalytic synthesis of 7-hydroxy-3- methyl coumarin.

#### 3.5.2. Removal of methylene blue (MB)

##### 3.5.2.1. Optimum conditions of the MB adsorption

###### 3.5.2.1.1. Effect of pH

**Fig. 6:**
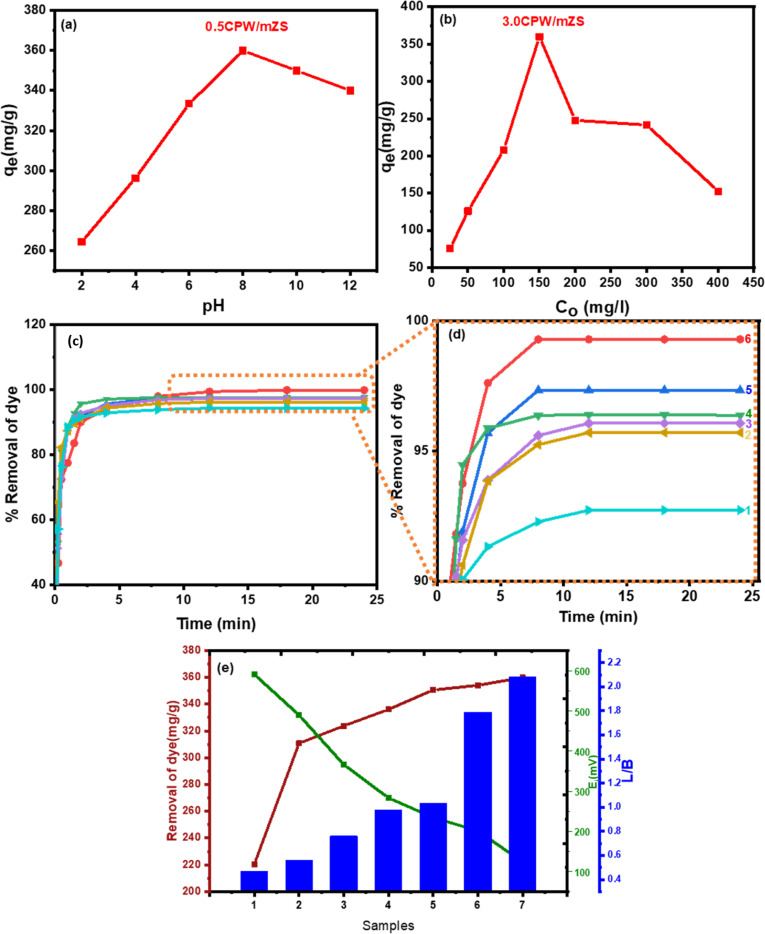
(a) pH effect, (b) initial concentration effect on MB adsorption by 3.0CPW/mZS (c) and (d) % removal of MB by (1) 0.5CPW/mZS, (2) 1.0CPW/mZS, (3) 1.5CPW/mZS, (4) 2.0CPW/mZS, (5) 2.5CPW/mZS, and (6) 3.0CPW/mZS, and (e) removal of dye (mg g^−1^), *E*_i_ (mV), and L/B ratio of (1) PW/mZS, (2) 0.5CPW/mZS, (3) 1.0CPW/mZS, (4) 1.5CPW/mZS, (5) 2.0CPW/mZS, (6) 2.5CPW/mZS, (7) 3.0CPW/mZS.

Fig. 6a depicted the effect of pH on the removal of MB from an aqueous solution; it was found that the optimal pH for MB adsorption from an aqueous solution is 8. For MB adsorption with an initial concentration of (150 mg L^−1^), the uptake capacity was significantly high in the basic medium compared to the acidic medium. The removal MB was gradually increased by pH increasing which reaches the maximum value at pH = 8. In an acidic medium, the lower uptake may be because of the partial protonation of the active sites which causes competition between H^+^ and MB^+^ ions at the active groups of adsorptions. This partial protonation blocked the active groups of the adsorbent surface, which prevented them from the complex formation with MB.^72^ The increase of solution pH up to 6 increased the ionization of the adsorbent with more negative charges on the adsorbent surface causing a decrease in the surface positive charge density. So, the electronic static repulsions between the adsorbent and the positively charged basic dye were less, thereby increasing the extent of adsorption. In particular, the initial concentration of the adsorbate has an impact on the dye uptake mechanism.

###### 3.5.2.1.2. Effect of MB initial concentrations

At optimum conditions for MB removal, 50 mL of test solutions with different initial concentrations (25–400 ppm) were studied. Fig. 6b showed that the maximum removal of dyes is at 150 ppm for MB. The adsorbent supplied specific sites for the dye to bind to; however, as the dye concentration increased, these specific sites became saturated, and the exchange sites caused by the large surface area of the adsorbent were occupied. It was evident that as initial concentrations of MB dye increase after the optimum concentration, the dye removal reduced.

###### 3.5.2.1.3. The removal of MB dye with contact time

Fig. 6c and d showed the variation of % removal of MB by Cs_*x*_H_3−*x*_PW_12_O_40_/mZS with the contact time at 150 mg L^−1^ initial MB concentration, pH = 8 and 30 °C, as evident at the start of the experiment, the high adsorption was attributed to the large external surface of the samples. Because the MB dye molecule does not need to go through the pores farther or deeper, it does not require a powerful driving force. Following that, the adsorption processes in the first section of curves in Fig. 6c and d, rose approximately linearly with contact time before plateauing. There are two stages to the MB dye adsorption kinetics process; the first stage is much faster than the second because there are a lot of active sites available for the adsorbate (MB). Even yet, the adsorption increased gradually until equilibrium was established as the number of saturated active sites increased. The approaching adsorbate was rejected by the exhausted binding sites over saturation, and the gradient of concentration between the solid surface and the solution was also altered. Previous research has stated that the adsorption could weaken with time as a result of just partially covering the adsorbent active sites.^73^

###### 3.5.2.1.4. Effect of surface acidity of Cs_*x*_H_3−*x*_PW_12_O_40_/mZS on the removal of MB

MB dye adsorption by Cs-PWA/mZS adsorbent took various forms and may be influenced by the increase in Lewis acid sites as evident from the ratio of Lewis/Brønsted acidity, the decrease in surface acid density/m^2^ and acid strength (*E*_i_), Fig. 6e and Table 2. As evident from Table 2 that the adsorption of MB by mZS, PW/mZS, 0.5CPW/mZS, 1.0CPW/mZS, 1.5CPW/mZS, 2.0CPW/mZS, 2.5CPW/mZS, and 3.0CPW/mZS increases with Cs content, which may be a result of a drop in acid strength (*E*_i_) and a rise in Lewis acid sites.^18^ Acids are electron pair acceptors according to the Lewis concept, whereas bases are electron pair donors. Meanwhile, the A–B complex (an addition compound or coordination compound) is created as a result of the primary reaction between Lewis acid A and Lewis base B.^74^ A coordination bond (also known as a dative bond or dipolar bond) is formed in this reaction between an electron-deficient atom of the acid and the base unshared electron pair. For Cs_*x*_H_3−*x*_PW_12_O_40_/mZS in solution is the Lewis base and MB would be essentially adsorbed as dimers (MB^+^)_2_ is the Lewis acid. Lewis acid–base interaction can result in a complex between Cs_*x*_H_3−*x*_PW_12_O_40_/mZS and the (MB^+^)_2_ dimer. The adsorption of MB was easily removed by the Cs salt of PW due to the substitution of PW protons by Cs ions, where the acid strength decreased and Lewis acid sites increased, increasing the adsorption capacity.

#### 3.5.3. Adsorption kinetic models

The most crucial factor for the ideal adsorption system at constant temperature and pH can be found by studying the adsorption isotherms, which describe the contact between adsorbent and adsorbate. The physicochemical parameters and the underlying thermodynamic assumption give us information about the adsorption mechanism, the surface characteristics, and the degree of adsorption affinity. The equilibrium amount of dye in the solution (mg g^−1^) *vs.* dye equilibrium concentration in the solution (mg g^−1^) was plotted to obtain the isotherm data. The following equations were demonstrated to express the Freundlich and Langmuir adsorption isotherms, eqn (S3) and (S4),† respectively, which may be used for both multilayer and heterogeneous surface adsorption.^2,75,76^

**Fig. 7:**
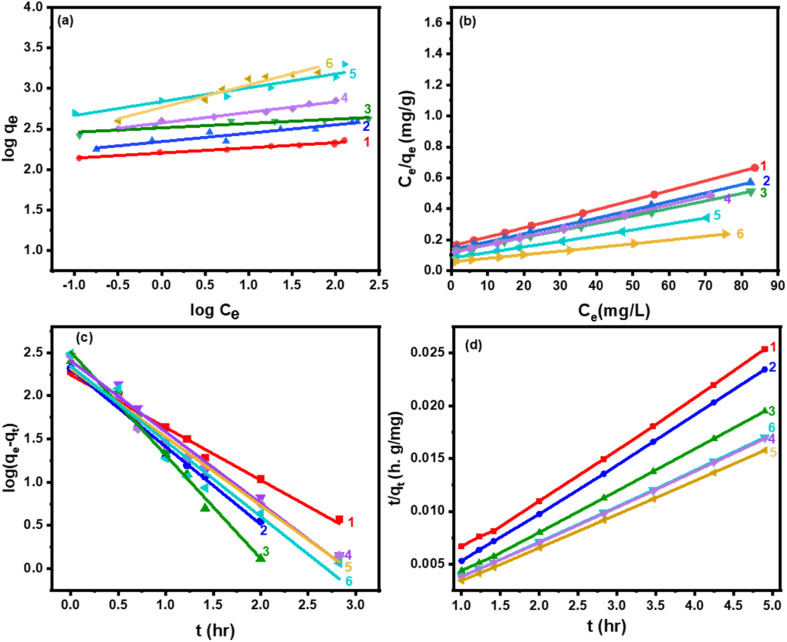
(a) Freundlich, (b) Langmuir isotherms, (c) 1st order, and (d) 2nd order kinetic models for the adsorption of MB on (1) 0.5CPW/mZS, (2) 1.0CPW/mZS, (3) 1.5CPW/mZS, (4) 2.0CPW/mZS, (5) 2.5CPW/mZS, and (6) 3.0CPW/mZS.

The data fit the Langmuir isotherms model well based on the results in Fig. 7a and b, and the maximum monolayer adsorption capacities (*q*_max_) of the catalysts were computed and given in Table 5. Moreover, one of the crucial elements of the Langmuir isotherm, the separation factor, *R*_L_, is given as:6
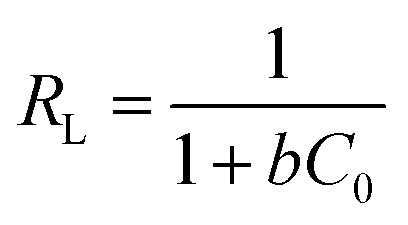
where *C*_0_ is the initial dye concentration (in mg g^−1^) in the liquid phase and *b* is the Langmuir constant. This parameter value shows whether the isotherm has an unfavorable shape (*R*_L_ > 1), a linear shape (*R*_L_ = 1), a favorable shape (0 < *R*_L_ < 1), or an irreversible shape (*R*_L_ = 0).^77^ For the adsorption on the as-synthesized adsorbents, *R*_L_ values were obtained and listed in Table 4, thereby demonstrating that MB benefited from the favorable adsorption. Moreover, the adsorption strength of the adsorbent is connected to the Freundlich constant (1/*n*). When 0.1 < 1/*n* ≤ 0.5, the dye is easily adsorbed onto the surface of the as-prepared catalysts. However, between 0.5 < 1/*n* ≤ 1, the process is somewhat challenging, while 1/*n* > 1 indicated the difficulty of the adsorption. The 1/*n* values of all adsorbents were shown in Table 4, demonstrating that MB could readily be adsorbed on the Cs-PWA/mZS surface. Besides, the isotherm parameters and the experimental values agreed, as evidenced by the high values of the regression coefficient (*R*^2^) (Table 4).

**Table 5:** Adsorption isotherm parameters of MB by the as-synthesized adsorbents

Samples	Freundlich isotherm			Langmuir isotherm		
	1/*n*	*K*_L_ (g^−1^)	*R* ^2^	*q*_max_ (mg g^−1^)	*R*_L_	*R* ^2^
0.5CPW/mZS	0.06	2.21	0.9426	311	0.0081	0.9991
1.0CPW/mZS	0.10	2.34	0.9394	324	0.0089	0.9990
1.5CPW/mZS	0.05	2.51	0.9424	336	0.0091	0.9989
2.0CPW/mZS	0.013	2.57	0.9774	351	0.0094	0.9988
2.5CPW/mZS	0.023	2.76	0.9307	354	0.0096	0.9992
3.0CPW/mZS	0.005	2.82	0.9776	360	0.0095	0.9987

On the other hand, the adsorption rate of MB was investigated using the linear form of the pseudo-first-order (1st order) kinetic model which is expressed as in eqn (S5)†:^78^

The mathematical expression for the pseudo-second-order (2nd order) kinetic model, which describes the chemical adsorption process and is based on the Langmuir adsorption isotherm, is as in eqn (S6)†:^79–81^

In Table 6, Fig. 7c and d, the parameters of the pseudo-first-order and pseudo-second-order sorption kinetics models are provided. The pseudo-second-order kinetic model, shows that the *q*_e,cal_ values, and the *q*_e,exp_ values exhibited excellent agreement. The adsorption of MB onto Cs-PWA/mZS was hypothesized to be a pseudo-second-order reaction, and this model better described the sorption kinetics of MB, suggesting that the bonding between the active sites of Cs-PW and MB dimer (MB^+^)_2_ may be responsible for the adsorption onto these surfaces. These findings revealed that the Cs-PW/mZS materials possessed enhanced uptake performance toward MB according to a high adsorption capacity and a quick adsorption process.

**Table 6:** Kinetic model parameters of MB by the as-synthesized adsorbents

Samples	*q*_e_ (exp.) (mg g^−1^)	Pseudo-first order			Pseudo-second order		
		*q*_e,cal_ (mg g^−1^)	*k*_1_ (h^−1^)	*R* ^2^	*q*_e,cal_ (mg g^−1^)	*k*_2_ (g mg^−1^ h^−1^)	*R* ^2^
0.5CPW/mZS	311	174	2.13	0.965	309	1.00 × 10^−5^	0.999
1.0CPW/mZS	324	204	2.25	0.974	323	0.96 × 10^−5^	0.998
1.5CPW/mZS	336	304	2.35	0.956	338	0.88 × 10^−5^	0.998
2.0CPW/mZS	351	302	2.45	0.977	348	0.83 × 10^−5^	0.999
2.5CPW/mZS	354	313	2.56	0.989	353	0.80 × 10^−5^	0.997
3.0CPW/mZS	360	321	2.62	0.978	360	0.77 × 10^−5^	0.999

#### 3.5.4. Mechanism of adsorption

##### 3.5.4.1. Intraparticle diffusion model

The rate-controlling stages and mechanisms were evaluated using the intraparticle diffusion model. According to the idea put by using Weber and Morris, who were the ones to initially establish the intraparticle diffusion model, the adsorption was, in essence, proportional to the square root of the contact time as shown by the following equation, where *k*_d_ is the diffusion coefficient:^82^

7 *q*_*t*_ = *k*_d_*t*^0.5^

**Fig. 8:**
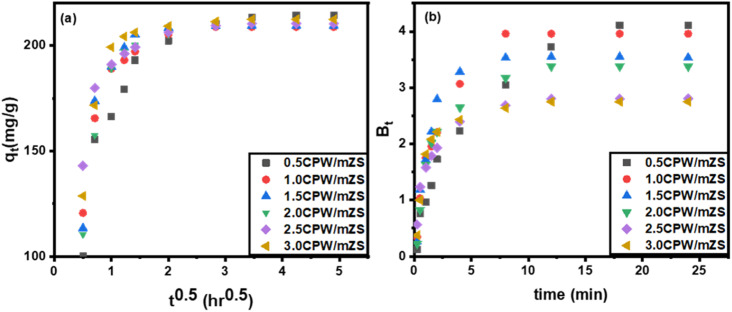
(a) Intraparticle diffusion and (b) Boyd plots for adsorption of methylene blue on CPW/mZS catalysts.

From Fig. 8a, plots of *q*_*t*_*versus t*^0.5^ of eqn (7), it was observed that the sorption process often involves two steps. It was discovered that a smooth curve ended the linear portion, which was then followed by another linear component. The sorption process appears to proceed by surface sorption and intraparticle diffusion, according to the two phases in the intraparticle diffusion plot. The first curved section of the plot (Fig. 8a) represented the boundary layer effect, and the second linear section represents intraparticle or pore diffusion. The origin was crossed by the plot of Fig. 8a, indicating that the intraparticle diffusion of MB into the channel mesopores of Cs_*x*_H_3−*x*_PW_12_O_40_/mZS is the only rate-controlling step, shown by external mass transfer (boundary-layer diffusion). It is therefore likely that MB is initially transported to the wider mesopores and then finally slowly diffused into small pores.

##### 3.5.4.2. Boyd kinetic model

The Boyd plot equation will help differentiate between intraparticle-transport-driven rates of adsorption and external transport because the twofold nature of the intraparticle diffusion plot indicated the presence of film and pore diffusion, eqn (S7)–(S9).†

In this study, we take *q*_e_ from the 2nd kinetic model (Table 6) and then

8 *B*_*t*_ = −0.4977 − ln(1 − *q*_*t*_/*q*_e_).

**Scheme 4:**
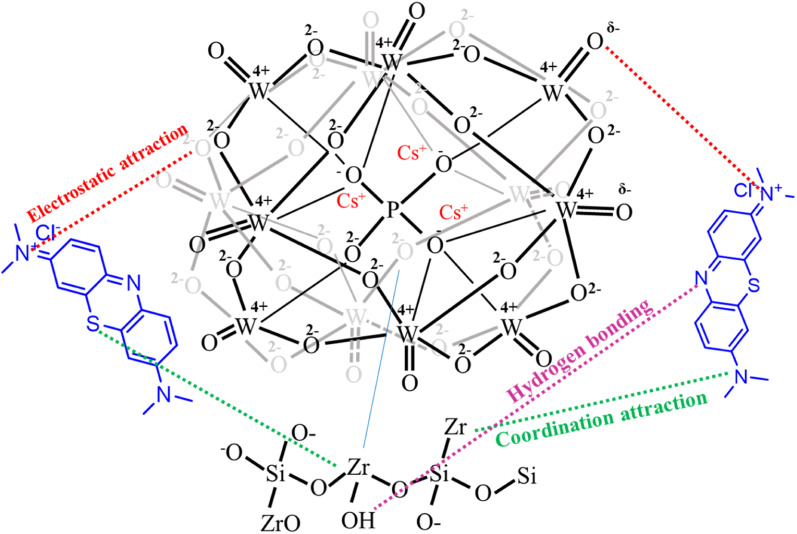
The proposed mechanism of the adsorption of MB dye on the surface of Cs_3_PW_12_O_40_/mZS.

To evaluate the external transport or intraparticle transport controls the rate of sorption, Fig. 8b of the plot *B*_t_ values *versus* time for MB dye at 30 °C is employed. The plots' linearity was demonstrated, but they did not cross the origin, demonstrating that external mass transfer dominates the sorption process and acts as the rate-limiting process at the start of the adsorption of MB dye in the examined adsorbent Cs_*x*_H_3−*x*_PW_12_O_40_/mZS.^76,83^ The obtained CPW/mZS substances are evaluated with other prior catalysts published for the production of MB Table S1.† The comparative research is based on the reaction parameters as well as the percentage removal of MB. Finally, the proposed mechanism for the adsorption of cationic dyes (MB) using Cs_*x*_H_3−*x*_PW_12_O_40_/mZS was illustrated in the Scheme 4. The figure showed a strong attraction between the donor atoms of the MB with the zirconium ion and also the electrostatic attraction and hydrogen bonding between the cationic functional groups of MB and negatively charged heteopoly acid.

## 4. Conclusion

In summary, Cs salt of tungstophosphoric acid supported mZS with varying extent of Cs ions content *x* (*x* = 0.5, 1, 1.5, 2, 2.5, and 3) have been prepared by two-step impregnation technique to increase the stability of the catalyst. The series of Cs salt of tungstophosphoric acid-modified mZS were investigated by XRD, FTIR, BET, and TEM. The surface of the samples was found to be affected by the Cs content. The B/L ratio of all samples was determined and showed that the increase of Cs salt content raised the Lewis acid sites. The catalytic activity of Cs_*x*_H_3−*x*_PW_12_O_40_/mZS was examined for production of the 7-hydroxy-4-methyl coumarin synthesis and decreased by the increase in Cs content of Cs_*x*_H_3−*x*_PW_12_O_40_/mZS. Moreover, the prepared samples are effective at removing hazardous dyes like MB from an aqueous solution. The amount of Cs content in the adsorbent increases the adsorption capacity. With a correlation coefficient of 0.998, the data from the adsorption equilibrium were found to suit the Langmuir equation. The results of the tests were in good agreement with the pseudo-second-order kinetic model, suggesting that the bonding between the active sites of Cs-PW/mZS and MB dimer (MB^+^)_2_ may play an important role during the adsorption technique. The intraparticle diffusion and Boyd plots of MB adsorption on Cs-PWA/mZS confirmed that the adsorption was mainly controlled by external mass transport which particle diffusion was the slowest step.

## Conflicts of interest

The authors declare no competing financial interest.

## Supplementary Material

RA-013-D3RA02235H-s001

## References

[cit1] Zhang X., Li Y., Xue L., Wang S., Wang X., Jiang Z. (2017). ACS Sustainable Chem. Eng..

[cit2] Ibrahim A. A., Salama R. S., El-Hakam S. A., Khder A. S., Ahmed A. I. (2021). Colloids Surf., A.

[cit3] Ibrahim A. A., Hassan S. M., Mannaa M. A. (2020). Colloids Surf., A.

[cit4] Eshghi H., Khojastehnezhad A., Moeinpour F., Bakavoli M., Seyedi S. M., Abbasi M. (2014). RSC Adv..

[cit5] Kozhevnikov I. V. (1998). Chem. Rev..

[cit6] Li X., Zhang Y. (2016). ACS Catal..

[cit7] Wen Y., Zhang H., Li J., An S., Chen W., Song Y.-F. (2021). ACS Sustainable Chem. Eng..

[cit8] Luzgin M. V., Stepanov A. G. (2014). J. Phys. Chem. C.

[cit9] Singh S., Patel A. (2014). Ind. Eng. Chem. Res..

[cit10] Kim H. , Jung J. C., Kim P., Lee K.-Y., Yeom S. H. and Song I. K., Scientific bases for the preparation of heterogeneous catalysts, Elsevier, 2006, vol. 162, pp. 801–808

[cit11] Marchal-Roch C., Laronze N., Villanneau R., Guillou N., Tézé A., Hervé G. (2000). J. Catal..

[cit12] Baranov A., Leonova L., Belmesov A., Domashnev D., Levchenko A., Shmygleva L., Karelin A., Dremova N., Dobrovolsky Y. (2022). Solid State Ionics.

[cit13] Abdollahi-Alibeik M., Pouriayevali M. (2011). React. Kinet., Mech. Catal..

[cit14] Kurhade A., Zhu J., Hu Y., Dalai A. K. (2018). ACS Omega.

[cit15] Mattos F. C. G. d., Carvalho E. N. C. B. d., Freitas E. F. d., Paiva M. F., Ghesti G. F., Macedo J. L. d., Dias S. C. L., Dias J. A. (2017). J. Braz. Chem. Soc..

[cit16] Lopes da Costa N., Guedes Pereira L., Mendes Resende J. V., Diaz Mendoza C. A., Kaiser Ferreira K., Detoni C., Souza M. M. V. M., Gomes F. N. D. C. (2021). Mol. Catal..

[cit17] Patel A., Narkhede N. (2012). Energy Fuels.

[cit18] Salama R. S., Hassan S. M., Ahmed A. I., El-Yazeed W. S. A., Mannaa M. A. (2020). RSC Adv..

[cit19] Ibrahim A. A., Ali S. L., Adly M. S., El-Hakam S. A., Samra S. E., Ahmed A. I. (2021). RSC Adv..

[cit20] Yuzbashi S., Mousazadeh M. H., Ramezani N., Sid Kalal H., Sabour B. (2020). Appl. Organomet. Chem..

[cit21] Khillare K. R., Aher D. S., Chavan L. D., Shankarwar S. G. (2021). RSC Adv..

[cit22] da Silva M. J., Rodrigues A. A., Lopes N. P. G. (2023). Chemistry.

[cit23] Ahmed A. I., Kospa D. A., Gamal S., Samra S. E., Salah A. A., El-Hakam S. A., Awad Ibrahim A. (2022). J. Photochem. Photobiol., A.

[cit24] Munir M., Nazar M. F., Zafar M. N., Zubair M., Ashfaq M., Hosseini-Bandegharaei A., Khan S. U.-D., Ahmad A. (2020). ACS Omega.

[cit25] Shakoor S., Nasar A. (2016). J. Taiwan Inst. Chem. Eng..

[cit26] Cheng J., Zhan C., Wu J., Cui Z., Si J., Wang Q., Peng X., Turng L.-S. (2020). ACS Omega.

[cit27] Al-Tohamy R., Ali S. S., Li F., Okasha K. M., Mahmoud Y. A. G., Elsamahy T., Jiao H., Fu Y., Sun J. (2022). Ecotoxicol. Environ. Saf..

[cit28] Weng C.-H., Pan Y.-F. (2007). J. Hazard. Mater..

[cit29] Sirirerkratana K., Kemacheevakul P., Chuangchote S. (2019). J. Cleaner Prod..

[cit30] El-Hakam S. A., Alshorifi F. T., Salama R. S., Gamal S., El-Yazeed W. S. A., Ibrahim A. A., Ahmed A. I. (2022). J. Mater. Res. Technol..

[cit31] Perren W., Wojtasik A., Cai Q. (2018). ACS Omega.

[cit32] Teh C. Y., Budiman P. M., Shak K. P. Y., Wu T. Y. (2016). Ind. Eng. Chem. Res..

[cit33] Altmann J., Ruhl A. S., Zietzschmann F., Jekel M. (2014). Water Res..

[cit34] Ali I., Basheer A. A., Mbianda X. Y., Burakov A., Galunin E., Burakova I., Mkrtchyan E., Tkachev A., Grachev V. (2019). Environ. Int..

[cit35] Lyu W., Li J., Trchová M., Wang G., Liao Y., Bober P., Stejskal J. (2022). J. Hazard. Mater..

[cit36] Zhu T.-T., Zhang Z.-M., Chen W.-L., Liu Z.-J., Wang E.-B. (2016). RSC Adv..

[cit37] Mahmoud M. E., Nabil G. M., Mahmoud S. M. E. (2015). J. Environ. Chem. Eng..

[cit38] Sonal S., Mishra B. K. (2021). Chem. Eng. J..

[cit39] Li H., Budarin V. L., Clark J. H., North M., Wu X. (2022). J. Hazard. Mater..

[cit40] Bakry A. M., Awad F. S., Bobb J. A., Ibrahim A. A., El-Shall M. S. (2020). RSC Adv..

[cit41] Rather I. A., Ali R. (2022). ACS Omega.

[cit42] Belluti F., Fontana G., Dal Bo L., Carenini N., Giommarelli C., Zunino F. (2010). Bioorg. Med. Chem..

[cit43] Li S., Qi X., Huang B. (2016). Catal. Today.

[cit44] Singh P., Kumar P., Katyal A., Kalra R., Dass S. K., Prakash S., Chandra R. (2010). Catal. Lett..

[cit45] Shimizu K.-i., Furukawa H., Kobayashi N., Itaya Y., Satsuma A. (2009). Green Chem..

[cit46] Abo El-Yazeed W. S., El-Hakam S. A., Salah A. A., Ibrahim A. A. (2021). J. Photochem. Photobiol., A.

[cit47] Suo H., Duan H., Chen C., Buffet J.-C., O'Hare D. (2019). RSC Adv..

[cit48] Cai Q., Luo Z.-S., Pang W.-Q., Fan Y.-W., Chen X.-H., Cui F.-Z. (2001). Chem. Mater..

[cit49] Chen L. F., Wang J. A., Noreña L. E., Aguilar J., Navarrete J., Salas P., Montoya J. A., Del Ángel P. (2007). J. Solid State Chem..

[cit50] Liu R., Zhang J., Xu Z., Zhao D., Sun S. (2018). J. Mater. Sci..

[cit51] Mohamed N. A. N., Han Y., Hector A. L., Houghton A. R., Hunter-Sellars E., Reid G., Williams D. R., Zhang W. (2022). Langmuir.

[cit52] Wang L., Yang J. (2022). Nanomaterials.

[cit53] Ibrahim A. A., Salama R. S., El-Hakam S. A., Khder A. S., Ahmed A. I. (2021). Colloids Surf., A.

[cit54] Pan G., Gu Z., Zhou Y., Li T., Gong H., Liu Y. (2011). Wear.

[cit55] Xiong R., Li X., Ji H., Sun X., He J. (2014). J. Sol-Gel Sci. Technol..

[cit56] Bertolini G. R., Pizzio L. R., Kubacka A., Muñoz-Batista M. J., Fernández-García M. (2018). Appl. Catal., B.

[cit57] Zhang W., Leng Y., Zhao P., Wang J., Zhu D., Huang J. (2011). Green Chem..

[cit58] Zhu X., Tian J., Liu X., Huang W., Luo D., Wang Z., Shan Z. (2017). RSC Adv..

[cit59] Kaipannan S., Marappan S. (2019). Sci. Rep..

[cit60] Pina-Salazar E. Z., Kaneko K. (2015). Colloids and Interface Science Communications.

[cit61] Nadaf S. N., Patil S. S., Kalantre V. A., Mali S. S., Hong C. K., Mane S. R., Bhosale P. N. (2020). J. Mater. Sci.: Mater. Electron..

[cit62] Blanchard J., Fajerwerg K., Breysse M., Beaunier P., Ribeiro M. F., Silva J. M., Massiani P. (2002). Catal. Lett..

[cit63] Abd El Rahman S. K., Hassan H. M. A., El-Shall M. S. (2012). Appl. Catal., A.

[cit64] Pizzio L. R., Vázquez P. G., Cáceres C. V., Blanco M. N. (2003). Appl. Catal., A.

[cit65] El-Yazeed W. S. A., Ahmed A. I. (2019). RSC Adv..

[cit66] Yaghoobi M., Zareyee D., Khalilzadeh M. A. (2020). Appl. Organomet. Chem..

[cit67] Maheswara M., Siddaiah V., Damu G. L. V., Rao Y. K., Rao C. V. (2006). J. Mol. Catal. A: Chem..

[cit68] Farahi M., Karami B., Keshavarz R., Khosravian F. (2017). RSC Adv..

[cit69] Tyagi B., Mishra M. K., Jasra R. V. (2008). J. Mol. Catal. A: Chem..

[cit70] Bennini-Amroun L., Mazari-Hachi T., Bouaziz-Terrachet S., Makhloufi-Chebli M., Rabia C. (2020). Chem. Data Collect..

[cit71] Lončarić M., Gašo-Sokač D., Jokić S., Molnar M. (2020). Biomolecules.

[cit72] Bhattacharyya K. G., Gupta S. S. (2008). Chem. Eng. J..

[cit73] Wang Y., Zhou Y., Jiang G., Chen P., Chen Z. (2020). Sci. Rep..

[cit74] Gal J.-F., Maria P.-C., Yáñez M., Mó O. (2021). Molecules.

[cit75] Morcos G. S., Ibrahim A. A., El-Sayed M. M. H., El-Shall M. S. (2021). J. Environ. Chem. Eng..

[cit76] Ediati R., Laharto P. B. F., Safitri R., Mahfudhah H., Oktavia Sulistiono D., Denisa Syukrie T., Nadjib M. (2021). Mater. Today: Proc..

[cit77] Li H., An N., Liu G., Li J., Liu N., Jia M., Zhang W., Yuan X. (2016). J. Colloid Interface Sci..

[cit78] Lata H., Garg V. K., Gupta R. K. (2007). Dyes Pigm..

[cit79] Pavan F. A., Mazzocato A. C., Gushikem Y. (2008). Bioresour. Technol..

[cit80] El-Hakam S. A., Ibrahim A. A., Elatwy L. A., El-Yazeed W. S. A., Salama R. S., El-Reash Y. G. A., Ahmed A. I. (2021). J. Taiwan Inst. Chem. Eng..

[cit81] Abo El-Yazeed W. S., Abou El-Reash Y. G., Elatwy L. A., Ahmed A. I. (2020). J. Taiwan Inst. Chem. Eng..

[cit82] Shen T., Gao M. (2019). Chem. Eng. J..

[cit83] Shu Y., Shao Y., Wei X., Wang X., Sun Q., Zhang Q., Li L. (2015). Microporous Mesoporous Mater..

