# Supplementary material for: Cesium salt of tungstophosphoric acid/mesoporous (zirconia–silica) composite for highly efficient synthesis of 7-hydroxy-4-methyl coumarin and removal of methylene blue
Source: RSC Adv. 2023 May 18;13(22):15243–60. doi: 10.1039/d3ra02235h (PMC10194047; doi:10.1039/d3ra02235h)
Supplement: RA-013-D3RA02235H-s001 [file RA-013-D3RA02235H-s001.pdf]

Electronic Supplementary Material (ESI) for RSC Advances.
This journal is © The Royal Society of Chemistry 2023

Cesium salt of tungstophosphoric acid/ mesoporous (zirconia-silica) composite for highly efficient synthesis of 7-hydroxy-4-methyl coumarin and removal of methylene blue

Amr Awad Ibrahim[a*], Doaa A. Kospa[a], O. R. Hayes[a], A. S. Khder[a,b], S. A. El-Hakam[a], Awad I. Ahmed[a*]

[a] Department of Chemistry, Faculty of Science, Mansoura University, Al-Mansoura 35516, Egypt.

[b] Chemistry Department, Faculty of Applied Science, Umm Al-Qura University, 21955 Makkah, Saudi Arabia

Tel.: +220502390551- E-mail: amr_awad@mans.edu.eg

$$n\lambda = 2d_{100} \sin(\theta) \qquad \text{equ S(1)}$$

$$a_0 = 2d_{100}/\sqrt{3} \qquad \text{equ S(2)}$$

$$\text{Log } q_e = \log K_F + \frac{1}{n} \log C_e \qquad \text{equ S (3)}$$

$$\frac{C_e}{q_e} = \frac{1}{K_L q_{max}} + \frac{C_e}{q_{max}} \qquad \text{equ S(4)}$$

Where K_F and n denote the Freundlich adsorption constants, q_m (mg/g) gives the monolayer adsorption capacity of the adsorbent, and K_L (L/mg) produces the Langmuir adsorption constant.

$$\ln (q_e - q_t) = \ln q_e - k_1 t \qquad \text{equ S (5)}$$

$$\frac{t}{q_t} = \frac{1}{k_2 {q_e}^2} + \frac{1}{q_e} t \qquad \text{equ S (6)}$$

Where the rate constants for the pseudo-first and pseudo-second order processes are k_1 (hr^{-1}), and k_2 (g/mg hr) are the rate constants for pseudo-first order and pseudo-second order reactions, respectively.

$$F = 1 - (6/\pi^2) \exp(-B_t) \qquad \text{equ S(7)}$$

$$B = \pi D / r^2 = \text{time constant} \qquad \text{equ S(8)}$$

r is the adsorbent particle radius, which is expected to be spherical, and F is the fraction of solute adsorbed at various times t, that D is the effective diffusion coefficient of the adsorbate in the adsorbent phase.

$$F = q_t/q_e \qquad \text{equ S(9)}$$

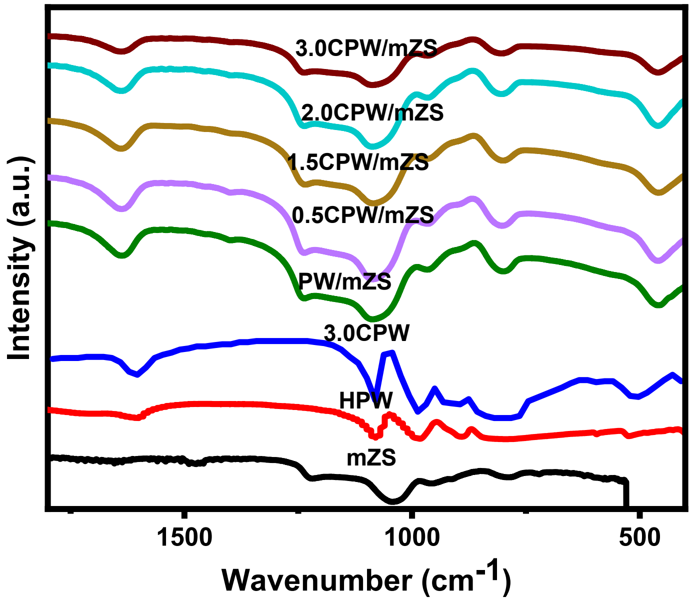

Fig. S1: FT-IR spectra of mZS, HPW, 3.0CPW, PW/mZS, and $Cs_xH_{3-x}PW_{12}O_{40}$/mZS samples from 1800- 400 cm^{-1}.

Adsorbents	C_o (MB) (mg/L)	Dye removal (%)	Adsorption capacity Q_{max} (mg/g)	References
Silk fibroin–graphene oxide (SF/GO)	100	96.0	381.7	1
Micro-mesoporous silica (MMZ)	100	91.0	303.1	2
12-tungestophosphoric acid/Zr/MCM-41 (25.0 wt% PTA/Zr-MCM-41)	--	--	259.1	3
2-amino-5-guanidinopentanoic acid modified activated carbon (AGDPA@AC)	60	75.0	219.9	4
Lysine and EDA double cross-linked graphene aerogel (LEGA)	150	97.5	332.2	5
H_3PO_4-modified corn stalks (P-CSs)	90	91.0	129.2	6
V_2CT_x MXene	50	94.8	111.1	7
nanosheet MFI zeolite (NZ)	---	---	476.2	8
Fava bean peels (FBP)	50	90.0	140.1	9
Fe-BDC MOF	5	94.7	8.6	10
CuO loaded Activated Carbon	50	99.2	10.5	11
**3.0 CPW/mZs**	**100**	**99.7**	**359.8**	**This work**

Table. S(1): Comparison study for removal of MB in other catalysts reported in the literature with this work.

1. L. J. Martis, N. Parushuram and Y. Sangappa, *Environ. Sci.: Adv.*, 2022, **1**, 285-296.

2. F. Subhan, S. Aslam, Z. Yan, M. Yaseen, M. Naeem, M. Ikram, A. Ali and S. Bibi, *Inorg Chem Commun*, 2022, **139**, 109380.
3. A. A. Ibrahim, R. S. Salama, S. A. El-Hakam, A. S. Khder and A. I. Ahmed, *Colloids and Surfaces A: Physicochemical and Engineering Aspects*, 2021, DOI: 10.1016/j.colsurfa.2021.127753, 127753.
4. M. Naushad, A. A. Alqadami, Z. A. AlOthman, I. H. Alsohaimi, M. S. Algamdi and A. M. Aldawsari, *J Mol Liq*, 2019, **293**, 111442.
5. L. Jiang, Y. Wen, Z. Zhu, X. Liu and W. Shao, *Chemosphere*, 2020, **265**, 129169.
6. Y. Tang, Y. Zhao, T. Lin, Y. Li, R. Zhou and Y. Peng, *Journal of Environmental Chemical Engineering*, 2019, **7**, 103398.
7. H. Lei, Z. Hao, K. Chen, Y. Chen, J. Zhang, Z. Hu, Y. Song, P. Rao and Q. Huang, *J Phys Chem Lett*, 2020, **11**, 4253-4260.
8. Y. Ji, F. Xu, W. Wei, H. Gao, K. Zhang, G. Zhang, Y. Xu and P. Zhang, *J Solid State Chem*, 2021, **295**, 121917.
9. O. S. Bayomie, H. Kandeel, T. Shoeib, H. Yang, N. Youssef and M. M. H. El-Sayed, *Sci Rep*, 2020, **10**, 7824.
10. C. Arora, S. Soni, S. Sahu, J. Mittal, P. Kumar and P. K. Bajpai, *J Mol Liq*, 2019, **284**, 343-352.
11. M. Ghaedi, A. M. Ghaedi, M. Hossainpour, A. Ansari, M. H. Habibi and A. R. Asghari, *Journal of Industrial and Engineering Chemistry*, 2014, **20**, 1641-1649.
